# An Overview of Molecular Modeling for Drug Discovery with Specific Illustrative Examples of Applications

**DOI:** 10.3390/molecules24091693

**Published:** 2019-04-30

**Authors:** Maral Aminpour, Carlo Montemagno, Jack A. Tuszynski

**Affiliations:** 1 Department of Chemical and Materials Engineering, University of Alberta, Edmonton, AB T6G 2R3, Canada; chancellor@siu.edu; 2 Ingenuity Lab, Edmonton, AB T6G 2R3, Canada; 3 Department of Oncology, University of Alberta, Edmonton, AB T6G 1Z2, Canada; jackt@ualberta.ca; 4 Southern Illinois University, Carbondale, IL 62901, USA; 5 Department of Physics, University of Alberta, Edmonton, AB T6G 2E1, Canada; 6 Department of Mechanical Engineering and Aerospace Engineering (DIMEAS), Politecnico di Torino, 10129 Turin, Italy

**Keywords:** molecular modeling, biomaterial, DNA sequencing, drug discovery

## Abstract

In this paper we review the current status of high-performance computing applications in the general area of drug discovery. We provide an introduction to the methodologies applied at atomic and molecular scales, followed by three specific examples of implementation of these tools. The first example describes in silico modeling of the adsorption of small molecules to organic and inorganic surfaces, which may be applied to drug delivery issues. The second example involves DNA translocation through nanopores with major significance to DNA sequencing efforts. The final example offers an overview of computer-aided drug design, with some illustrative examples of its usefulness.

## 1. Introduction to Molecular Modeling Methods

Computer experiments play an increasingly significant role in science today. The advent of high-performance computing has enabled virtual experimentation in silico as a tool which allows for interpolation between laboratory experiments and theory. Schulten introduced the term “computational microscope” to describe the role of computational simulations in augmenting experimental research when direct measurements are not possible. He believed that computational biophysics has progressed to the point where it presents a realistic view of intra-cellular components, often at a resolution not attainable through laboratory instruments, reaching atomic or even electronic dimensions [1]. Feynman presciently stated in 1964: “Certainly no subject or field is making more progress on so many fronts at the present moment than biology, and if we were to name the most powerful assumption of all, which leads one on and on in an attempt to understand life, it is that all things are made of atoms, and that everything that living things do can be understood in terms of the jigglings and wigglings of atoms” [2]. Molecular dynamics (MD) is an important computational tool for understanding the physical basis of the structure, the dynamic evolution of the system, and the function of biological macromolecules. Fourteen years later, the first MD simulation of a biological macromolecule, namely, Bovine pancreatic trypsin inhibitor (BPTI), was published [3]. Although the relatively accurate X-ray structure of BPTI was available at the time, its physiological function was unknown. 

One of the most significant resources for MD simulations is the Research Collaboratory for Structural Bioinformatics (RCSB, www.rcsb.org), which makes available 3D experimentally-determined biological macromolecular structural data. The RCSB Protein Data Bank (PDB) is a worldwide repository for processing and distribution of 3D structure data of macromolecules, such as proteins and nucleic acids [4], and is an essential resource for biomolecular modeling. X-ray crystallography has made the largest contribution to our understanding of protein structure. Crystallization for many proteins (e.g., membrane proteins) is a difficult task that may require considerable effort. An important measure of the accuracy of a crystallographic structure, meanwhile, is its resolution. Relaxing a protein structure using molecular mechanics (MM) energy minimization methods reduces the energy of the protein structure and leads to a subtly different structure in important ways. In high-resolution structures, the only dynamic property that can be obtained is the isotropic temperature factor (B factor or Debye factor). Nuclear magnetic resonance (NMR) is another method by which to determine the protein structure. In NMR, the magnetic spin properties of atomic nuclei are used to build up a list of distance constraints between atoms in an enzyme. This list is then used to build a model of the protein that shows the location of each atom. A major advantage of NMR spectroscopy is that it provides information on proteins in solution, as opposed to those forming a crystal. Direct determination of structure by NMR is generally restricted to smaller proteins, typically under 20 kDa in molecular weight. High-resolution X-ray powder diffraction has also been used to solve and refine protein structures [5]. This method shares the advantage of not requiring a protein crystal. It should be noted that the structures obtained from experiments require some post-processing to prepare them for simulations. Issues could be encountered due to missing residues, atom clashes, crystallographic waters, and alternate locations that should be resolved before MD simulations are performed. In structure preprocessing, missing residues and missing atoms such as hydrogen are added, and atomic clashes are eliminated. Ionization, protonation states, and tautomer states are assigned as well to ensure the best quality structure to start.

Quantum mechanics (QM) methods (e.g., ab initio molecular orbital or density functional theory calculations) can be used to study reactions for molecular systems consisting of hundreds of atoms. Biological systems are particularly challenging for ab initio quantum mechanics methods because of their large sizes, yet first principles calculations can now tackle problems of great biological interest that cannot be solved by other means. In this regard, a detailed atomistic investigation of a biological system requires knowledge of its electronic structure. For instance, enzymatic reactions involve bond-forming and bond-breaking, effects that need to be treated quantum-mechanically [1]. Quantum calculations can provide useful models of transition states and reaction intermediates. Another example relates to photoreceptors (such as rhodopsin [6,7,8] and involves excited states and the interaction between the biomolecules and the electromagnetic field. QM-based approaches may also be instrumental in correctly describing polarization effects (e.g., in ion channels [9]) as well as metallo-proteins, where very subtle chemical phenomena (such as the fact that the metal ion ligand bond has a partially covalent nature) play a vital role. Finally, QM calculations are useful in comparing different variety of spectroscopic data, such as infrared spectroscopy (IR) [10,11], Raman [12,13] and NMR [14], which can be obtained from the electronic structure calculations without additional empirical assumptions [15]. Direct applications of first principle approaches to the study of biomolecules are still restricted to systems of up to a few hundred atoms, while the size and conformational complexity of biological systems calls for methods capable of treating up to several 100,000 atoms over time scales of tens of nanoseconds. 

Hybrid quantum mechanics/molecular mechanics (QM/MM) simulations significantly expand the scope of quantum mechanical calculations to much larger systems by partitioning the problem into two parts, each of which is treated with different computational methods. The part of the system directly participating in a given chemical reaction, such as catalysis, involves the active site, substrates, and directly participating amino acid residues and is treated with a QM level simulation. The remaining part of the enzyme, which does not participate directly in the reaction and typically encompasses a much larger number of atoms, is simulated using molecular mechanics with a biomolecular force field (FF). QM/MM methodologies can differ notably in terms of features, such as: (1) the type of scheme used to calculate the QM/MM energy; (2) the different boundary regions chosen; (3) how the interaction between the QM and MM region is investigated; (4) how an appropriate computational method is selected; and (5) how the enzymatic reaction and the associated conformational flexibility are tackled. There are advantages and disadvantages to these different QM/MM methods, largely depending on the type of enzyme and reaction under study. The most common QM/MM methods are Car–Parrinello/Molecular Mechanics MD [16], empirical valence bond (EVB) Method [17], the cluster model [18], and QM/MM MD Methods. 

MD uses Newton’s equations of motion to determine the net force and acceleration experienced by each atom from which it simulates the time evolution of a set of interacting atoms. Each atom i at position r_i_ is treated as a point with a mass m_i_ and a fixed charge, q_i_. The atomic coordinates evolve according to the laws of Newtonian physics where $F_{i}$ is the force exerted on the mass m_i_ and $a_{i}$ is the acceleration of it, i.e.:

(1) $$F_{i}=m_{i}a_{i}$$

⇓

(2) $$F_{i}\left(t\right)=m_{i}\frac{d^{2}r_{i}\left(t\right)}{dt^{2}}$$

F*_i_*(*t*) can also be expressed as the gradient of the potential energy, where *V* is potential energy (i.e., FF).

(3) $$F_{i}=-\nabla_{i}V$$

⇓

(4) $$-\nabla_{i}V=m_{i}\frac{d^{2}r_{i}\left(t\right)}{dt^{2}}$$

The engine of an MD program, it should be noted, is its time integration algorithm. The most popular integration methods for MD calculations are Verlet, velocity Verlet, and Leap-Frog Algorithms [19]. In the Verlet algorithm, two third-order Taylor expansions are used for the positions r(t), one forward and one backward in time. Denoting velocity as v, acceleration as a, and the third derivative of r(t) with respect to t as b, we obtain the following expression:

(5) r(t+Δt)= r(t)+ v(t) Δt+(12)a(t)Δt2+ (16)b(t)Δt3+O(Δt4)

(6) r(t−Δt)= r(t)− v(t) Δt+(12)a(t)Δt2− (16)b(t)Δt3+O(Δt4) 

Adding the two expressions gives:

(7) $$r\left(t+\Delta t\right)=\;2r\left(t\right)-r\left(t-\Delta t\right)+a\left(t\right)\;\Delta t^{2}+O\left(\Delta t^{4}\right)$$

Accordingly, a(t) is the force divided by mass, such that:

(8) a(t)=−(1m) ∇V (r(t))

The time step used in MD calculations is approximately one order of magnitude smaller than the fastest motion (hydrogen molecule’s bond vibration), which is about 10 femtoseconds (fs). 

The result of an MD simulation is a trajectory in a 6*N*-dimensional phase space (3*N* positions and 3*N* momenta). However, such a trajectory is usually not particularly relevant in and of itself. MD is a statistical mechanics method and that generates a set of configurations distributed according to some statistical distribution function, or also known as a statistical ensemble. Three different ensembles are commonly used in MD simulations: the Microcanonical Ensemble (NVE), the Canonical ensemble (NVT), and the Isotherma-isobaric ensemble (NPT). These ensembles are used during equilibration to achieve the desired temperature and pressure before changing to the constant-volume or constant-energy ensemble when data collection starts. Here, N stands for the number of particles, E for energy, V for volume, and P for pressure. Each of these denotes a value to be kept constant during simulation. Consequently, measuring quantities in MD usually entails performing time averages of physical properties over the system trajectory (averages over configurations). For instance, one can define the instantaneous value of a generic physical property *A* at time *t* as:

(9) $$A\left(t\right)=f\left(r_{i}\;\left(t\right),\;\ldots ,\;r_{N}\left(t\right),\;v_{i}\left(t\right),\;\ldots ,\;v_{N}\left(t\right)\right)\;$$

We then obtain its average as:

(10) $$\langle A\rangle =\;\frac{1}{N_{T}}\;\overset{N_{T}}{\underset{t=1}{\sum }}A\left(t\right)\;$$

where *t* is an index which runs over the time steps from 1 to the total number of steps, *N_T_*.

Sufficiently long simulation times ensure that the phase space will be well sampled, such that the averaging process approximates the corresponding thermodynamic properties. The most commonly measured properties are potential energy, kinetic energy, total energy, temperature, pressure, and root-mean-square deviation (RMSD). RMSD is the measure of similarity between structures and is found as the average distance between the atoms (usually the backbone atoms) of superimposed proteins. Therefore, the lower RMSD is, the better the model will be in comparison to the target structure. RMSD is found as:

(11) $$RMSD=\;\sqrt{\frac{1}{N}}\overset{N}{\underset{i=1}{\sum }}\delta_{i}^{2}.\;$$

where *δ_i_* is the distance between atom, *i*, and a reference coordinate. This quantity is often calculated for the backbone heavy atoms or in some cases just the *C_α_* atoms.

The heart of any MD scheme is the FF used to analytically describe the atomistic interactions. The atomic forces that govern molecular movement can be divided into those caused by interactions between atoms that are chemically bonded and those caused by interactions between atoms that are not bonded.

(12) $$E_{total}=\;E_{bonded}+\;E_{nonbonded}\;$$

(13) $$E_{bonded}=\;E_{bond}+\;E_{angle}+E_{dihedral}\;$$

(14) $$E_{nonbonded}=\;E_{electrostatic}+\;E_{van\;der\;waals}\;$$

In other words, Force fields present the potential energy surface of the system represented by a closed set of analytical potential energy functions. Since the kinetic energy in force fields is also taken into account, the system is able to move across the energy barriers on the potential energy surface, which implies substantial changes (e.g., conformational) during the simulation. The results of simulations will be realistic only if the potential energy function mimics the forces experienced by the ‘real’ atoms. 

**Figure 1:**
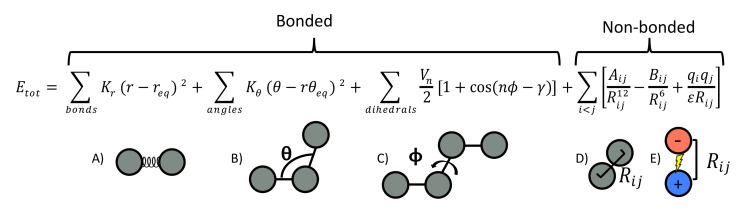
A typical force field (FF) model. Energy dependencies are related to (a) stretching or compressing a bonded pair of atoms (modeled by a simple spring) (b) Increasing or decreasing the bond angle (modeled by a simple spring) (c) dihedral angle rotations (modeled by a sinusoidal function) (d) Van der Waals interactions (modeled by Lennard–Jones potential) and (e) Electrostatic interactions (modeled by Coulomb’s law). (a–c) are caused by interactions between atoms that are chemically bonded to one another while (c–e) are caused by interactions between atoms that are not bonded.

An example of an equation used to approximate the atomic forces that govern molecular movement is depicted in Figure 1. 

In a typical FF, chemical bonds and atomic angles are modeled using simple springs (quadratic energy functions) that do not allow bond breaking. The functional form for dihedral energy is highly variable, so dihedral angles (that is, rotations about a bond) are modeled using a sinusoidal function that approximates the energy differences between eclipsed and staggered conformations. Additional improper torsional terms may be added to impose the planarity of aromatic rings, and “cross-terms” may be used to describe coupling of different internal variables, such as angles and bond lengths. Some FFs also include explicit terms for hydrogen bonds. Non-bonded forces arise due to van der Waals interactions and are modeled using the Lennard–Jones potential, and charged (electrostatic) interactions are modeled using Coulomb’s law. 

There are major limitations present in all FFs, one being the lack of polarizability, since each atom is fixed, forbidding any change in polarizability over time. However, both non-bonded terms can be scaled by a constant factor to account for electronic polarizability and produce better agreement with experimental observations. Although polarizable FFs are very promising, they remain computationally very expensive, with their use and parameterization being less user-friendly than that of their fixed charge counterparts [20]. Another major limitation of FFs is that they cannot be used to study reactivity, since bonds cannot be broken or formed during the simulation. To overcome this, the aforementioned QM/MM methods can be employed. ReaxFF [21] is one of the emerging methods in the study chemical reactivity using classical MD. ReaxFF enables chemical reactions to be studied through a geometry-dependent parameterization of reactants and products. Since chemical reactions are not permitted in simulations using conventional FFs, certain protonation and tautomeric states must be assigned in advance to all system residues and maintained during the simulation. 

FF parameters are determined from experiments in physics, chemistry, and/or electronic structure calculations (using QM). A set of parameters is defined for different types of atoms, chemical bonds, dihedral angles, and so on. For example, an FF would include distinct parameters for an oxygen atom in a carbonyl functional group and in a hydroxyl group. A typical parameter set includes values for atomic masses, van der Waals radii, and partial charges for individual atoms; equilibrium values of bond lengths, bond angles, and dihedral angles for pairs, triplets, and quadruplets of bonded atoms; and values corresponding to the effective spring constants for each potential. For example, a typical FF for propane contains 10 bond-stretching terms, 18 angle-bending terms, eighteen tortional terms, and 27 non-bonded interactions [22]. Some popular FFs used in classical MD simulations are AMBER, CHARMM, and GROMACS.

Biologically relevant macromolecules, such as proteins, do not operate as static, isolated entities. Contrarily, they are involved in numerous interactions with other species in such a highly specific manner and recognition. Binding between two interacting systems has both enthalpic (ΔH) and entropic (-TΔS) components and is associated with a negative Gibbs free energy of binding (ΔG = ΔH-TΔS), which may have differing thermodynamic signatures, varying from enthalpy- to entropy-driven. Thus, the understanding of the forces driving the recognition and interaction require a detailed description of the binding thermodynamics, and a correlation of the thermodynamic parameters with the structures of interacting partners [23]. More on free energy methods will be discussed in Section 4. 

### Length and Time Scale Limitations in Molecular Dynamics Simulations

**Figure 2:**
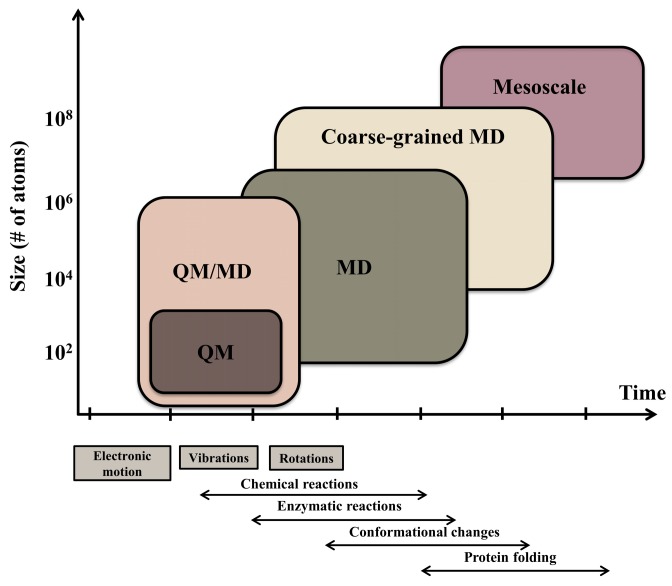
Spatiotemporal resolution of molecular modeling techniques: The temporal (abcissa) and spatial (ordinate) resolutions of each technique are indicated by colored boxes. The timescales of some fundamental molecular processes, as well as composite physiological processes, are indicated below the abcissa.

Large system sizes and long timescales are two challenges frequently encountered in MD simulation. Moreover, MD simulations are computationally demanding for two reasons. First, the force calculation at each time step requires considerable computation. Second, the force calculation must be repeated many times. For example, to produce sufficient sampling to study folding, 1012 timesteps are required to reach millisecond timescales. Individual steps are limited to a few femtoseconds (fs ~10−15 s), so simulating a millisecond of physical time requires nearly one trillion timesteps. Most biomolecular events of interest, such as protein folding, protein-drug binding, and major conformational changes essential to protein function, typically take longer (microseconds to milliseconds) than the MD timescales, thus limiting the applicability of these simulations. Spatiotemporal resolution of molecular modeling techniques is illustrated in Figure 2. 

MD simulations have been performed on large macromolecular systems such as the ribosome [24,25] or entire viral capsids of viruses such as satellite tobacco mosaic [26,27] and HIV1 [28]. Moreover, protein folding simulations [29,30] and protein dynamics and functions [31] for smaller systems have been simulated for 10–100 μs. The largest photosynthetic membrane simulation published thus far is that of a 100-million-atom MD simulation of a chromatophore from *Rb. sphaeroides* [32]. Various methods have been used to overcome the size and timescale limitations in MD. The coarse graining (CG) method simplifies and accelerates MD simulations [33,34,35,36]. CG employs mesoscale models, in which a group of atoms is treated as a single interaction site or a bead, this idea having been introduced by Levitt and Warshel in the 1970s [37,38]. Enhanced sampling methods also address the timescale issue, and these include Steered molecular dynamics (SMD), Umbrella sampling (US) [39], and Metadynamics [40]. US [39] is one notable equilibrium-collective variable-based enhanced sampling method, while SMD [41,42] and metadynamics [41] are the most popular nonequilibrium ones [43]. SMD has been used to accelerate the biomolecular simulations by applying external forces. It has been extensively used to calculate the potential of mean force along aquaporin channels. SMD has also been used to mimic forces that naturally arise in the context of atomic force microscopy (AFM) and optical tweezer experiments [44,45,46,47,48,49], and can be used to drag the ligand along the possible pathways predicted from electrostatic surface potential in drug design simulations [43]. 

The US pioneered the use of enhanced sampling methods. An energy term or a bias potential, mostly harmonic potential, is applied to the system along a reaction coordinate, and moves it from its initial state to its final state by varying, for example, the forces, distances, and angles manipulated in the simulation. MD, meanwhile, can be used to simulate the intermediate states. The weighted histogram analysis method (WHAM) is the most popular postprocessing method, and it analyzes a series of umbrella sampling simulations [50]. WHAM is performed by unweighting and stitching together the underlying free energy function, leading to a potential of mean force (PMF) reconstruction. This methodology has been successfully applied to numerous drug discovery-relevant problems [51].

Metadynamics is a relatively new MD-enhanced sampling technique to efficiently sample the phase space and map out the underlying free energy landscape as a function of collective variables. Here, a history-dependent repulsive bias potential as a function of a set of collective variables is added to the Hamiltonian of the system in order to push the system away from its local energy minima. This can be achieved through the addition of a small Gaussian-shaped potential to the current bias to encourage the system to explore the regions of the phase space that are otherwise not sampled by conventional MD by escaping from a saddle point to a nearby local minimum, where the procedure is repeated. When all minima are occupied with Gaussians, the system diffuses to a barrier-free state along the collective variable, and the simulation can be stopped. 

Finding a set of collective variables (coordination numbers, the number of hydrogen bonds, relative molecule orientation/rotation and bond lengths, angles or torsions) is a challenging task in Metadynamics simulations. Recently, metadynamics has been combined with docking calculations to study a number of ligand–target complexes, demonstrating the power of this method to characterize binding and unbinding paths, to treat conformation flexibility, and to compute free-energy profiles without requiring an excessive computational cost [52]. 

Among other challenges in MD simulations, like other simulation techniques, is the heavy computational time and production of the large data. The amount of data necessary to represent a rigid biomolecule (~100Kb) can increase 5 orders of magnitude (~10Gb) when simulated. As simulations have greatly increased in scale reaching cellular levels, the large amount of data generated by computer simulations intrinsically presents big data challenges. System sizes between 50,000 and 1 M atoms are common nowadays for simulations of single macromolecules or macromolecular complexes [53]. Recently, Molecular dynamics simulations have been extended to highly crowded heterogeneous cellular systems (100 M atoms for the cytoplasm of a bacterial cell) and simulations of entire cells in molecular detail will soon become reality [54]. The time scales covered by such simulations are now routinely reaching 1 µs and in exceptional cases as much as 1 ms [55]. Depending on how often coordinates are saved, this means that a single simulation may generate data on terabyte to petabyte scales. The large amounts of data coupled with the high degree of complexity in many systems presents formidable challenges in managing, analyzing, and interpreting such big data in comparison with experiments that are being discussed. As traditional approaches to the analysis of simulations do not scale well to highly complex systems of macromolecules, a greater emphasis on automated machine learning and artificial intelligence will be required in the future [56,57]. On the other hand, the increased capabilities and flexibility of recent modern graphics processing units (GPUs) hardware combined with high level GPU programming languages such as CUDA and OpenCL has made computational power accessible to computational community. Many molecular modeling applications are well suited to GPUs since they adopt themselves to he design and implementation of data-parallel algorithms that scale to hundreds of tightly coupled processing units. One of the most time consuming calculations in a typical molecular dynamics simulation is the evaluation of forces between atoms that do not share bonds. The high degree of parallelism capability of GPUs can attain performance levels twenty times that of a single CPU core. The twenty-fold acceleration accessible by the GPU decreases the runtime for the non-bonded force evaluations such that it can be overlapped with bonded forces and PME long-range force calculations on the CPU. According to the latest Molecular dynamics GPU benchmarking report provided by NAVIDA-Tesla (https://nvidianews.nvidia.com), compared to CPUs, GPUs run common molecular dynamics, quantum chemistry, visualization, and docking applications more than 5 times faster.

In the era of petascale computing, large-scale MD simulations are having a profound impact in numerous and diverse scientific endeavors [58]. These have ranged from the treatment of disease and development of drugs [59,60] to biotechnological applications such as the fabrication of novel biomaterials [61], DNA sequencing [62], and the creation of bio-based renewable energy sources [63]. Two notable applications of biomolecular modeling have been in bionanotechnology and structure-aided drug design fields. For bionanotechnology applications we discuss below two rapidly developing computational areas: organic–inorganic interface simulations to design smart novel materials, and modeling nanopores for DNA sequencing purposes. 

## 2. Organic–Inorganic Interface Simulations for Smart Novel Material Discoveries

Hybrid organic-(bio)-inorganic materials play a major role in the development of advanced functional nanomaterials with nanobiotechnological applications. The development of these materials represents an emerging interdisciplinary topic at the interface of biology, material science, and nanotechnology. It was Thompson, in his book, *On Growth and Form* [64], who first described in detail the complex nature of inorganic crystal morphologies formed in association with living organisms and the presence of a pre-existing template controlling the growth of inorganic material. The mineralization process is usually controlled by biomacromolecules such as proteins and peptides, and the resulting hybrid structures have a physiological function. Examples of naturally occurring hybrid materials include seashells, bone, teeth, and cartilage. Adsorption and assembly of biomolecules on material surfaces through interactions on a nanometric scale form the basis for the preparation of bio-nanohybrid materials. The effects of selective adsorption of biomolecules in an active inorganic material is of critical importance in many applications, motivating the development and optimization of active surfaces for biosensors [65], bioactive nanoparticles [66], biocatalysis [67], bioanalytical systems for diagnostics and detection [68], solar cells [69], and bioseparations. Biomaterials have been designed for use in advanced drug delivery systems for over 60 years. Biomaterials for drug delivery are engineered to protect and then release molecules at desired rates [70]. Drug delivery systems has its own chemical, physical and morphological characteristics, and may have affinity for different drugs polarities through chemical interactions (e.g., covalent bonds and hydrogen bonds) or physical interactions (e.g., electrostatic and van der Waals interactions) [71]. All these factors influence the interaction of nanocarriers with biological systems [72]. To achieve optimal outcomes in drug delivery systems different parameters, such as the composition of the nanocarriers (e.g., organic, inorganic, and hybrid materials) and the form in which drugs are associated with them (such as core–shell system or matrix system) are also fundamental for understanding their drug delivery profile. 

Current experimental methods are unable either to track the trajectory and follow the dynamics of bio-material interactions at the picosecond scale or to observe the surface morphology and growth at the nanoscale level. Computational modeling, however, has accelerated the discovery of hybrid materials by providing in-depth understanding at the interface between organics and inorganics and by reducing the experimental trials. 

The rapidly growing interest in bio-material interfaces, particularly for bionanotechnology applications, necessitates proteins or peptides designed to recognize the inorganic surface with high specificity. Molecular modeling and simulation methods ranging from quantum mechanics, to atomistic, and coarse-grained simulations have been employed to investigate protein–surface interactions at different levels of time and length scales.

**Figure 3:**
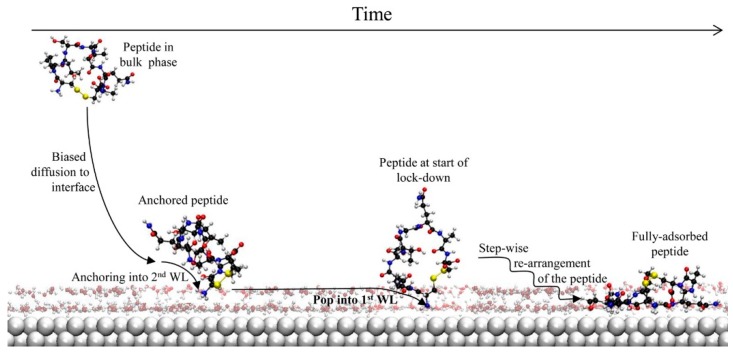
This illustration shows the simulations of proteins adsorbed on different surfaces. [74]. The first step is the biased diffusion towards the interface. The second step is the ‘anchoring’ of the peptide via a hydrophilic group of the peptide to the second water layer (WL) that occurs adjacent to the solid surface and finally the third step is the formation of the fully adsorbed peptide through a (‘lockdown’) process of stepwise rearrangement of the peptide that is initiated by the anchor group popping into the WL immediately adjacent to the surface.

Penna and Biggs [73] offer up significant new molecular-level insight into the adsorption mechanism by considering results from over 240 MD simulations of up to 100 ns length in which one of two different peptides adsorb after starting from a distance beyond the range of the peptide-solid surface interaction. They asserted that their proposed peptide adsorption mechanism at the molecular level was generalizable for the case where the interaction between the surface and the solution above it is strong, such as would occur for metal surfaces. They proposed a putative three-phase adsorption mechanism as illustrated in Figure 3: (1) biased diffusion of protein or peptide from bulk solution towards the interface; (2) ‘anchoring’ of the peptide or protein via a hydrophilic group of the peptide to the second water layer (WL), which is adjacent to the solid surface; and finally (3) formation of the fully adsorbed peptide, which occurs through a (‘lockdown’) process of stepwise rearrangement of the peptide that is initiated by the anchor group popping into the WL immediately adjacent to the surface [74].

**Figure 4:**
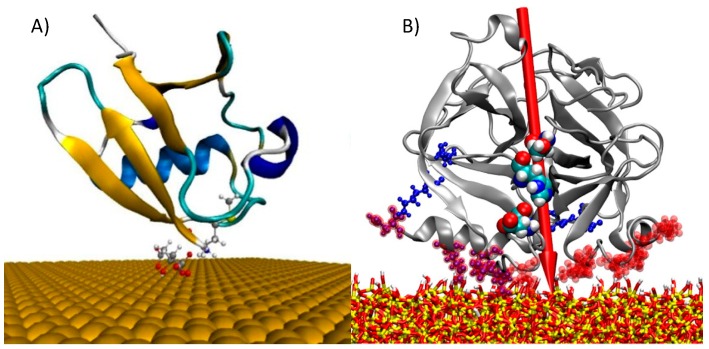
Modeling of proteins on different surfaces (**a**) ab initio Quantum Mechanics (QM), Molecular Dynamics (MD) and Brownian dynamics is used to study Ubiquitin-Au complex [75]. (**b**) The adsorption of α-chymotrypsin and hen egg white lysozyme on amorphous silica is studied by means of MD simulations in comparison with adsorption experiments. [76].

Simulating the interactions of peptide–inorganics requires a comprehensive understanding of protein and the surface properties, such as surface morphology, size, shape, chemical composition, and adsorption characteristics. Most commonly studied surfaces interacting with proteins and peptides are metals and metal oxides due to their applications in nanobiotechnology. Peptide interactions with metals have been extensively studied due to their inertness (e.g., noble metals) and various applications in sensors, bioelectronic devices, medical implants, and catalysts. Exploiting the driving forces for molecular recognition, crystal growth, and shape development promises to yield a useful strategy for material design and performance predictions. Two examples of inorganic–organic models are shown in Figure 4. 

While atomistic simulations and FF parameters are well established for describing biomolecules and inorganic materials, FF parameters of these simulations related to one side may not be applicable for the other one, and they are likely to fail for the interfaces. Finding the right dataset to parameterize an FF for organic–inorganic hybrid systems is a challenging task, but it is necessary for geometry optimization and quantitative calculation of system properties. Another significant challenge in modeling of organic–inorganic interactions is the unavailability of FFs and software that adequately describe the inorganic surface, the biomolecule, and the cross-terms. The INTERFACE FF [77] currently includes the PCFF-INTERFACE, CHARMM-INTERFACE, and CVFF-INTERFACE distributions that can be used with Discover, Forcite (Materials Studio), LAMMPS [78], and NAMD [79]. Alternative FFs also include GOLP [80] and GOLP-CHARMM [81] FFs to account for polarizability. These models fail when metal atoms are not fixed and thus they are mostly applicable to idealized surfaces. 

**Figure 5:**
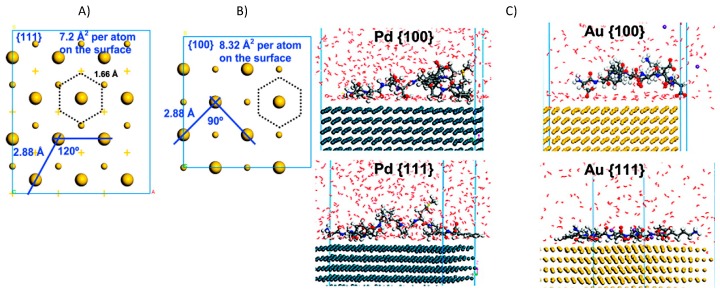
Comparison of the geometry (**a**) of the (111) surface and (**b**) of the (100) surface with numerical values for Au. Larger spheres represent the top atomic surface layer, smaller spheres represent the hcp sites and the crosses represent the fcc sites. Dotted black lines indicate energetically favorable orientations of aromatic rings such as in Tyr, Phe, and Trp. In (**a**), the ring or a sp2-hybridized group can occupy free fcc (111) “lattice” sites which contributes to high binding strength, while this is difficult to achieve in (100) lattice. Therefore, the small, flexible solvent molecules and possibly designed molecules with a rectangular repeat are of higher binding strength. (**c**) Representative snapshots of the Flg−Na_3_ peptide on the pd and Au metal surfaces. Water interlayer is maintained between the peptide and the (100) surface, resulting in lower adsorption energy [85].

A large number of computational studies have been performed on the interface of proteins/peptides and metals such as Au(111), Au(100), Pd, Pt and Ni and Pd-Au bimetal [82,83,84]. Heinz et al. optimized Lennard Jones (12-6 and 9-6) parameters for several face-centered cubic (fcc) metals such as gold, palladium, and platinum and their interfaces with organic and inorganic molecules, water, and biopolymers, and thereby achieved the ability to accurately reproduce densities, surface energies, and interface energies. Furthermore, based on MD simulations and experimental results, it has been concluded that the mechanism of adsorption conforms to soft epitaxy (a crystalline overlayer on a crystalline substrate) for peptides on metal surfaces [85]; this mechanism is characterized by the coordination of polarizable atoms (C, N, O) in peptides with epitaxial fcc, and epitaxial hexagonal cubic packing (hcp) sites on the metal surface. Therefore, the molecular size and geometry, rather than the specific chemistry, determine the adsorption energy. Independent of the type of metal, simulations have shown a preference in adsorption of peptides on fcc(111) facets over (100) facets. This preference can be explained in terms of the available fcc lattice cites above the metal surface. A hexagonal spacing of ~ 1.6 A between available lattice spacing favors aromatic rings, while a quadratic spacing of ~2.8 A prefers only small molecules such as water (see Figure 5a.). Large molecules, such as common sp2 (Arg, Trp, Gln, Tyr, Asn, and PPh3) and hybridized sp3 in peptides adsorb most strongly since they are a very good fit to (111) metal surfaces, while short molecules with sp3 hybridized alkyl groups exhibit the least attraction. The phenyl ring of hexagonal symmetry, for example, can best coordinate on (111) surface (flat-on parallel conformation) rather than on (100) and (110) surfaces (tilted conformation). The strength of adsorption of peptides on metal surfaces is a result of competition between small water molecules with the peptides (see Figure 5b). Residues such as Phe, Arg, Tyr, Trp, His, and Asp (F, R, Y, W, H, and D) that contribute to binding are in direct contact with the metal surfaces, and, in contrast, less-binding residues are separated from the surface by one or two water layers, thus resulting in lower adsorption energy. 

According to simulations, the binding differential of the peptides to (111) and (100) facets in the soft epitaxial adsorption can lead to the selective stabilization of crystal facets during nanoparticle growth from seed crystals, while various phenylalanine containing peptides are employed as shape directing templates for nanoparticles. It has been shown that the presence of the phenyl ring in peptide sequences with and without F is sufficient as a molecular switch to convert cuboctahedral or cubic nanocrystals into tetrahedral during the growth process [86]. In particular, decorated metal nanoparticles have applications in biomining [87], cell targeting, imaging, and therapeutic purposes [88,89]. Specific binding of peptides on graphene [90,91,92,93,94] has also been investigated both computationally and experimentally. Graphene and Graphite surfaces are hydrophobic in nature, which results in negative binding energy of peptides in aqueous solutions. Common residues with higher affinity for graphene and graphite involve H, Y, W, and F, as well as amide groups in Q and N [90,91,92,93,94,95]. The epitaxial interaction between graphite layers is expected to be weak (the cleavage energy is only 190 mJ m^−2^). Therefore, the possibility of pi-stacking interactions contributes to moderate adsorption of aromatic molecules.

SiO2 [96,97,98,99] and TiO2 [100,101,102] have been widely studied due to their numerous potential applications. Silica is a crucial component in drug carriers [103,104], catalyst support [105] and filler in deformed modifier of polymer composites and hydrogels [106]], and is one of the most abundant oxides on Earth. Developing FFs for the interface between silica and water is very challenging due to the variety of the surface chemistry and the complex nature of the interface. Several FFs have been developed for bulk silica [107,108], silicon oxides [109], and alpha-quartz [110]. Rimola et al. developed FFsiOH FFs for both bulk and surface silica [111]. Ramakrishnan et al. parametrized the existing CVFF FF with silicon parameters for n+silicon (1 0 0) surface [93,112], and studied the specific binding of peptides selected by phage display on it. Strongly bonded peptides on n+ Si(100) surface, meanwhile, have been identified as M, W, D, T, H, S, and R [93]. The surface chemistry of the amorphous silica particles is, therefore, dependent on the surface topography; the distribution of Q4, Q3, and Q2 environments; and the distribution of siloxane (Si–O–Si), silanol (Si–OH), and ionic siloxide (Si–O–···Na+) groups. 

Adsorption on oxide surfaces is governed by a different mechanism than are metals such as ion pairing, hydrogen bonds, hydrophobic interactions, and conformational changes of peptides. It has been shown that the ion pairing is the dominant mechanism of peptide binding when the surface charge of silica is significant. A higher pH value in the solution results in a higher negative charge density on the silica surface, and favors the adsorption toward positively-charged peptides, while it weakens the attraction of negatively charged peptides and reduces the influence of hydrogen bonds and hydrophobic interactions. pH has little effect on neutral peptides, which are not as strongly bound by hydrogen bonds and hydrophobic interactions. Interfacial hydrogen bonds involve oxygen and hydrogen atoms in silanol groups, siloxide ions, and lattice oxygen atoms on the silica surface in contact with alcohol groups, backbone amide groups, and aromatic hetrocycles in peptides. Hydrogen bonds play a primary role when the charge of the silica is zero for all peptides, and peptides with cationic groups can predominantly adsorb to silica surface at any pH. In addition, Peptides containing hydrophobic residues increasingly interact with the silica surface. Residues such as F, W, L, I, and V can be effectively drawn to silica surfaces at lower pH, as well as to silica surfaces of lower area density of silanol groups. The interaction of hydrophobic residues originates form depletion forces to avoid disruptions of the network of hydrogen bonds in the aqueous phase that would occur when the hydrophobic residues remain immersed. As a result, there is no intrinsic attraction of these groups to silica, and the driving force is rather the exclusion from water on less ionized silica surfaces. On increasingly ionized silica substrates, hydrophobic groups do not approach the surface because they would disrupt the hydration shells of siloxide ions and of cations in proximity of the surface. Another contribution to the adsorption mechanism arises from the conformation preference of the peptides on silica surfaces, especially for longer sequences of peptides. The effect of conformational changes on the adsorption mechanism is more dominant in metal oxides compared to the metals as the reduction in surface energy in metal oxides [113,114]. 

Titanium oxide (Titania) surfaces exhibit ionization of superficial terminal hydroxyl groups (Ti-oh) similar to silica, and have exhibited similar mechanisms of molecular recognition and binding. Steered MD simulations show that the ion pairing is the dominant binding mechanism between TiO- groups and positively charged R and K residues in the sample peptide (RKLPDA). The free energy profile of RKLPDA on an oxidized titanium surface has been calculated using metadynamics and replica exchange with solution tempering (REST). The typical conformations for the peptide on the surface involves flat as well as upright conformation with R and K residues bounded to the surface [61]. Furthermore, Monti et al. and Carravetta have parametrized the FF for the rutile TiO2(110) [101,115].

Computer simulation of material surfaces and interfaces is still in the early stages of development, and most of the studies related to the interactions of surface and interface have been limited to electrostatic and Van der Waals interactions. However, the interactions occurring at surfaces and interfaces are not restricted to non-bonding interactions. These interactions constitute a complex process including adsorption, diffusion, and even breaking or forming covalent bonds. Some specific methods have been developed to meet such demands. ReaxFF, for instance, was developed to allow molecular bonds to be broken and re-formed and covalent chemical reactions to take place in a simulation without using quantum mechanics. [21,116], while the QM/MM method was developed to meet the demands both of serving large simulation systems and ensuring high accuracy [117,118]. 

## 3. Modeling of Nanopores for DNA Sequencing Applications

DNA sequencing technology provides an opportunity to identify the genetic risk factors associated with complex human diseases, and has the potential to establish a new era of personalized medicine in medical research and health care. The chain-termination method, proposed by Sanger and Gilbert in 1977 [119], was the first feasible method by which to detect nucleic acid sequences. However, according to the National Human Genome Research Institute, it costs at least $10 million to sequence the enormous amount of 3 billion base pairs of the DNA found in the genomes of humans using Sanger-based sequencing. The growing need for faster and more cost-effective sequencing of the human genome has created enthusiasm for the development of new technologies that surpass the conventional Sanger chain-termination methods in terms of speed and economy. Nanopore sensors are promising DNA sequencing technologies poised to meet this demand. The basic idea of using nanopores for DNA sequencing was proposed by Church et al. in 1995 [120]. The basic idea is to disperse either double-strand (dsDNA) or single-strand (ssDNA) in a salt solution and apply an electric field in order to pass the DNA molecule through the pore. As the DNA translocates through the pore, the ionic flow will be blocked. Therefore, by monitoring the changes in ionic current as a function of time, it should be possible to determine which nucleotide or base is in the nanopore at the time. Despite multiple efforts and some promising developments, though, several crucial issues in DNA nanopore sequencing, such as the high speed of the translocation (temporal resolution) and the low single-nucleotide sensitivity of the ionic current (spatial resolution), have not yet been fully resolved. Experimental challenges encountered in DNA nanopore sequencing have stimulated significant computational studies to resolve these experimental issues. The computational investigations can directly relate the microscopic state of the system to the measured current, providing an important molecular picture of the mechanisms of DNA sequence detection in a manner not attainable through experimentation. MD simulations also provide a means to study the translocation of biological molecules through nanoscale pores at levels of detail that are difficult to achieve with experimental methods alone [121,122,123,124,125]. Biological, solid-state, and the combination of both (hybrid) nanopores can be used for DNA sequencing. α-haemolysin and Mycobacterium smegmatis porin A (MspA) membrane pores, it should be noted, were the first biological pores to demonstrate the feasibility of DNA sequencing experimentally. 

α-Hemolysin (α-HL) was the first and is most commonly used biological nanopore, representing a remarkable value in the field of DNA sequencing. Several properties of this mushroom-shaped heptamer make this membrane channel suitable for various biotechnological applications. First, the structure of α-HL remains functionally stable at temperatures close to 100 °C [126] within a wide pH range (pH 2–12) [126]. Furthermore, the inner diameter of the α-HL channel and the size of a single-stranded DNA (ssDNA) molecule are very close in size (diameter ~ 1.3 nm), such that the α-HL nanopore is able to discriminate single nucleotides using the ionic current inside the nanopore [127]. Since its transmembrane pore is open at normal conditions [128], α-hemolysin has the ability to spontaneously bind to various biological or synthetic lipid bilayers [129], and does not require specific ionic conditions. It has been experimentally observed that the interaction between ssDNA and the α-HL pore channel depends strongly on the orientation of the ssDNA molecules with respect to the pore. Remarkably, the voltage-free diffusion of the 3′-threaded DNA (in the trans-to-cis direction) is two times slower than the corresponding 5′-threaded DNA having the same poly(dA) sequence. All-atom molecular dynamics simulations of this system delineate a microscopic mechanism for the asymmetric behavior. In a confining pore of biological protein, the ssDNA straightens and its bases tilt toward the 5′ end, assuming an asymmetric conformation. Consequently, the bases of a 5′-threaded DNA experience larger effective friction and forced reorientation that favors co-passing of ions. MD simulation results denote that the translocation process through a narrow pore is more complicated than previously believed and involves base tilting and stretching of ssDNA molecules inside the confining pore. It is worth noting that MD simulations have been performed without any a priori knowledge of the experimental data, (i.e., which orientation is faster or causes larger ion current blockade) [130].

**Figure 6:**
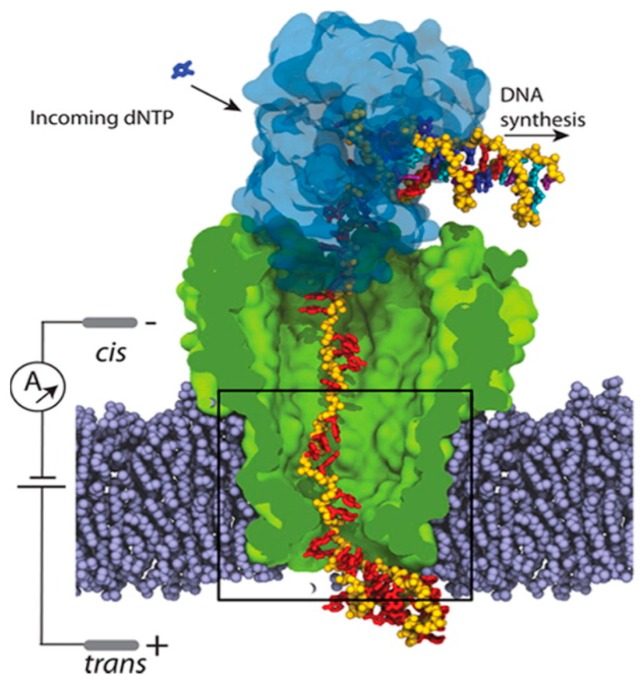
Molecular dynamics simulations of blockade currents in biological nanopores: All-atom model of MspA (green) suspended in a lipid bilayer membrane (purple spheres). A phi29 polymerase (blue semitransparent surface) is bound to the junction of single- and double-stranded DNA (the DNA backbone is represented by the yellow spheres [132]. The water molecules and ions are not shown in either figure.

Another commonly used biological nanopore is MspA. It has been experimentally shown that DNA strands immobilized inside the MspA pore produce ionic current blockades that permit identification of a single nucleotide substitution in the DNA sequence [131](see Figure 6). However, with a high transport velocity of the DNA within the nanopore, the ionic current cannot be used to distinguish signals within noise. The Aksimentiev group investigated, through all atom molecular dynamics (MD) simulations (~100 μs in total), the molecular origin of such extreme sensitivity of the ionic current to the sequence and orientation of DNA strands. The constriction of this nanopore, they found, contains positively asparagine substitutions. They performed several arginine mutations to examine the effect of the positively-charged residues on the DNA transportation speed, discovering that additional positively charged arginine substitutions near the constriction of MspA can cause a ~10- to 30-fold reduction in DNA translocation speed due to continuous contact between the arginine substitution and the DNA backbone, or due to base-stacking interactions while preserving the nucleotide-type specificity of the ionic current blockades. These results reveal the importance of nanopore geometry and charge location and may help to direct the modification of nanopores such as MspA to improve their utility for nanopore sequencing and other nanopore technologies. The same group coupled a MspA biological nanopore with a DNA processing enzyme, which opens the double-stranded DNA helix and slowly ratchets one of its strands through the pore channel in order to reduce the speed of DNA transportation through the MspA protein. Employing molecular dynamics simulations, they found that the quantity of the displaced water from the nanopore by the DNA strand regulates the nanopore ionic current, whereas the steric and base-stacking properties of the DNA nucleotides control the amount of water displaced. Unexpectedly, they found the effective force on DNA in MspA to undergo large fluctuations, which may produce insertion errors in the DNA sequence readout [132]. 

Despite having shown much promise, the sensitivity of the lipid membrane used to fix biological nanopores to the temperature, pH value, and salt concentration, and the applied bias have been identified as major drawbacks with respect to the use of biological pores in practical applications. For this reason, solid-state nanopores, fabricated in membrane materials such as SiO2, Si3N4, Al2O3, and plastic have emerged as a promising alternative to biological nanopores [133,134,135], as they not only are robust and durable within the given environment but also permit convenient, controllable, and reproducible manipulation of physical and chemical properties of nanopores, in addition to delivering the advantage of being readily integrated into semiconductor devices and chips. 

There have been extensive studies conducted on double-stranded DNA (dsDNA) translocation [136,137,138,139,140,141,142,143,144,145,146,147,148], single-stranded DNA (ssDNA) translocation [138,149], and protein translocation [150,151] through solid-state pores. A wealth of interesting results have been obtained with respect to solid-state nanopores, such as translocation time as a function of DNA length [137], salt dependence on ion transport during DNA translocation [146,147,152], unzipping of DNA during translocation [141,153], and discrimination of ssDNA and dsDNA based on pore diameter [138,154]. However, the thickness of solid-state nanopores makes it difficult to detect individual base-specific modulation in ion currents as multiple base pairs interact with the nanopore channel simultaneously [155]. Single-layer materials such as graphene [133,134,135,156], MoS2 [157], and BN [138,158,159] nanopores have been used as an alternative to conventional solid-state pores. A potential advantage of these ultrathin membranes is their thickness (~0.3 nm, which is comparable with the height of the nucleotide), which improves spatial resolution in sequencing measurements. Instead of actually building and testing the device experimentally, molecular dynamics simulations can assist and enable a bottom-up design of two-dimensional material nanopore devices by unveiling the atomic-level processes occurring during nanopore sensing.

Graphene is one of the most fascinating single-layer materials for DNA sequencing. It is impermeable to ions, and, due to its strength, it can form a freestanding membrane, enabling the ideal atomically thin membrane for nanopore measurements. Another important and advantageous property of graphene is that it is electrically conductive, which opens up the possibility to monitor an in-plane current through the membrane when the DNA molecule translocates. However, despite intensive research efforts in this area, the identification of individual bases has not yet been achieved by graphene nanopores, mainly because the speed of DNA translocation through the pore is too high to permit individual bases to be distinguished. Furthermore, the conformational fluctuations of DNA inside the pore add significant noise to the measured signal. Computational simulations have been conducted to investigate whether indeed DNA sequencing is possible with ionic current detection through graphene nanopores. Sathe et al. studied the interaction of DNA with the pore while DNA translocates through a graphene pore in order to evaluate the manner in which this affects the ionic current [160,161,162]. Initially, they found that poly(AT) and poly(GC) can be distinguished at a bias voltage of 1 V [160]. However, the simulations also exposed some complications with the approach, as they showed that the bases move stochastically through the pore, leading to sequencing errors. Furthermore, the blockage in the current was predicted to be highly dependent on the local conformation of the DNA bases inside the pore, resulting in a strong overlap of the current blockades for the different bases [160,161]. Interestingly, hydrophobic adhesion of bases to the graphene surface right next to the pore was found to considerably reduce the possible single-stranded DNA conformations, leading to a reduction in the translocation speed of the DNA through the nanopore [161]. These simulations suggested that a three-layer graphene sheet produces the best ‘stepwise’ translocation pattern, such that collective binding and unbinding of the bases on both sides of the membranes is possible, while fluctuations in the DNA base conformations inside the pore are minimized [161]. 

**Figure 7:**
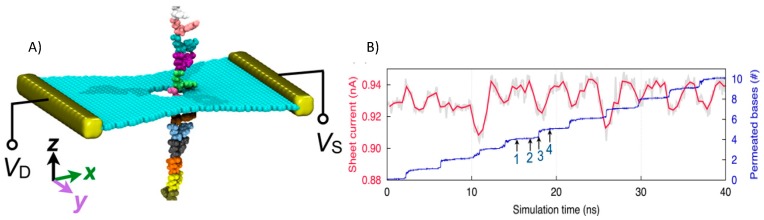
Electronic detection of stepwise motion of stretched ssDNA through graphene nanopore: (**a**) Schematic of ssDNA and a graphene nanopore, and (**b**) Transverse calculated sheet current through graphene shown together with the number of permeated nucleotides during ssDNA translocation [163].

Recently, quantum mechanics Green’s function-based transport calculations were used to calculate the transverse electronic conductance of the graphene sheet while the stretched ssDNA are translocating through the pore to record the intrinsic stepwise DNA motion (see Figure 7). The measurement scheme described paves the way to enhance the signal-to-noise ratio, not only by slowing the DNA translocation to provide sufficient time for base recognition, but also by stabilizing single DNA bases and thereby reducing thermal noise [163]. 

To overcome the limitations of biological and solid state nanopores, it has been proposed to combine the best properties from both types of nanopores [164,165], leading to next-generation hybrid nanopores. The idea is to attach specific biological recognition groups inside the solid-state nanopores. This should substantially improve the chemical specificity, while still maintaining mechanical and chemical stability. Several hybrid nanopores have been created [164,165,166,167]; however, there are no reports in the literature of their having been used in DNA sequencing.

In summary, nanopore-based DNA sequencing offers an inexpensive, reliable technology which could thrust genomics into personal medicine and open a new frontier in gene detection. Instead of physically building and testing the DNA sequencing device experimentally, with the associated cost, molecular dynamics simulations can be employed to build a bottom-up design of biological or material nanopore devices. Simulation unveils the atomic-level processes occurring during nanopore sensing, guiding experiments to overcome significant challenges in the effort to reach single-base resolution, given the fast translocation times, the conformational fluctuations, the stochastic translocation of the bases, and the high noise levels. To address the abovementioned challenges, alternative materials can be used for nanopore structures because we can evidently not change the properties of the DNA. Furthermore, the nanopore geometry (pore diameter and charge on the pore surface) can greatly modulate the drag forces, something that is not considered in most simulations. In addition, the error rate of DNA sequencing has not been assessed in most computational studies since the read lengths of the DNA molecules used in these studies have been very short. All these issues need to be attended in the future.

## 4. Computer-Aided Drug Design 

One notable application of biomolecular modeling is Computer-Aided Drug Design (CADD). Drug discovery begins with target and lead discovery, followed by lead optimization and pre-clinical in vitro and in vivo studies to identify the best candidate compounds that satisfy the main criteria for drug development. [8] Drug design using in silico methods is cost-effective compared to in vivo and in vitro methods and it may also reduce the time it takes for a drug to reach the market. In this regard, an article in the October 5, 1981, issue of *Fortune*, entitled the “Next Industrial Revolution: Designing Drugs by Computer at Merck” (Van Drie, 2007 [168]), is sometimes referred to as heralding the inception of the field of computer-aided drug design (CADD).

**Figure 8:**
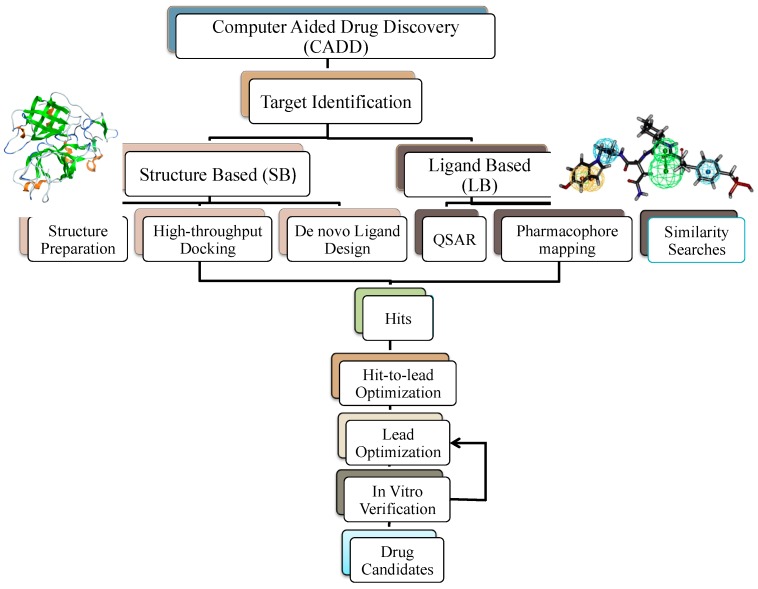
Schematic representation of a Computer-Aided Drug Design (CADD) pipeline whose methods are mainly classified into structure-based and ligand-based methods. Hits are identified, filtered, and optimized to obtain potential drug candidates to be experimentally tested [52].

CADD methods can be broadly classified into two groups, namely, ligand-based (LB) and structure-based (SB) drug discovery (see Figure 8). When the target structure is not experimentally determined (via X-ray Crystallography or NMR) or when it is found to be challenging to predict a structure using homology modeling or ab initio methods, ligand-based approaches are often used as an alternative. Also, LBDD methods can be performed even if the target structure is available. These methods, however, rely on information about known actives. Molecular similarity approaches, QSAR (quantitative structure–activity relationship) modeling, and pharmacophore modeling are some popular LBDD approaches. In LBDD molecular similarity approaches, common structural features of ligands (fingerprints) that bind to a target are used to carry out the screening [169]. The similarity search approach allows for the representation of a molecule in such a way that it can be effectively compared against other molecules [170]. Another notable approach is the quantitative structure–activity relationship (QSAR), which models the relationship between the structural features of the ligands that bind to a target and the corresponding biological activity effect [171]. Another field gaining ground in the area of computational ligand-based drug discovery is pharmacophore modeling, where common structural features of ligands that bind to a target are used to carry out the screening [172]. The 3D structure of various drug molecules, it should be noted, is now available in several large databases such as NIH [173], ZINC [174,175], and DrugBank [176]. 

In SBDD methods, the 3D structure of the target (protein receptors, enzymes) is known and can usually be obtained by X-ray crystallography or NMR experiments as mentioned above. If the crystal structure of the target is not known, then homology modeling can be used to build the 3D structure of the target. Knowing the target structure makes it possible to exploit effective tools such as structure-based virtual screening and direct docking methods on targets and possible drug molecules. In structure-based virtual screening, large databases of chemical structures are searched in order to identify the potential drug candidates that are most likely to bind to a drug target. In this process, the drug candidate will be docked into a protein target, and a scoring function will be applied to estimate the probability that the drug candidate will bind to the protein target with high affinity [177]. Geometry-based binding site identification algorithms can be used to predict the binding pocket sites of the protein. Binding pocket sites indicate the location where small molecules can bind to target structures, which are associated with diseases. In this regard the high affinity drug candidates are called “hits”. Molecular docking is a method that can predict the preferred orientation of one molecule (ligand) to a second (receptor, protein) when they bind and form a stable complex structure [178]. *De novo* ligand design is another method that makes it possible to build up ligand molecules that are drug-like within the constraints of the binding pocket by assembling small pieces in a stepwise manner with much less search space having to be explored. The biochemical and organic model builder (BOMB) program, as a *de novo* ligand design method, can be used to design ligands that bind to the target without using ligand databases by adding substituents into a core structure of ligand molecules [179]. Inhibitors for *E. coli* RNS polymerase, for instance, have been designed using this method [180]. 

High affinity hit molecules that have passed through the hit-to-lead optimization process are called leads. During lead optimization, the effectiveness of hits obtained is augmented to reach the desired affinity, drug safety, and absorption, distribution, metabolism, and excretion/elimination (ADME) properties. For more precise results, the affinity of hit molecules to targets can be evaluated by computing various estimates of binding free energies. The outcome selected lead molecules are tested in vitro for their activity. Lead optimisation is, by its very nature, an iterative process whereby the information between In vitro verification and lead optimization is interchanged, leading to candidate drug. This narrows down the drug candidates and allows experimentalists to focus their resources on examining compounds likely to have a given activity of interest. 

Structure-based virtual screening processes are fast but are associated with an underlying problem, which is the plasticity of the target. In most cases the flexibility of the target molecules is limited or ignored. MD simulations are frequently used in drug design processes, and can be used to generate multiple receptor conformations for virtual screening purposes. Multiple snapshots can be extracted from MD trajectory as a different conformation variant of the binding pocket representative. MD can also be used as a post-docking tool to validate and/or refine docking solutions. In this case, MD is able to distinguish the bad docking poses from meaningful ones. First, the complex ligand-receptor structure is not stable and the ligand may even leave the binding site. In this regard, RMSD analysis can be used to check the stability of the ligand-receptor structure. MD in explicit solvent, meanwhile, can take into account the presence of structural water molecules within the binding site, which is important for correctly predicting ligand binding [181]. MD simulation is also an important tool in identifying the drug binding pathway for a number of reasons; first, insight into the binding pathway is crucial in gaining a thorough understanding of ligand binding kinetics [182]. Second, it is important for investigation of non-active site mutations which could possibly prevent drugs from entering binding sites by rupturing the structure of the binding pathway. Finally, the intermediate states in the pathway of a ligand may themselves be additional binding sites to be considered in drug development. [183]. Enhanced sampling methods are often coupled to MD simulations to expedite the drug-binding processes and to extract useful thermodynamic and kinetic data. A number of techniques have been developed in this regard, among them free energy perturbation (FEP), umbrella sampling, SMD, and metadynamics, all of which have been briefly described above. 

One of the thermodynamic observables that can be computed from MD trajectory is free energy. Given that the free energy of a system is a function of its state, the free energy difference found between the initial and final (binding/unbinding) states is independent of the path taken between them. Differences in binding energies can be evaluated by different methods. Free energy perturbation and thermodynamic integration are among the most popular free energy methods [184,185,186]. Relative binding affinity of a drug-target system can be calculated using a technique called “alchemical transformation”, although in drug design they are known as “free energy perturbation” (FEP) methods [187]. 

**Figure 9:**
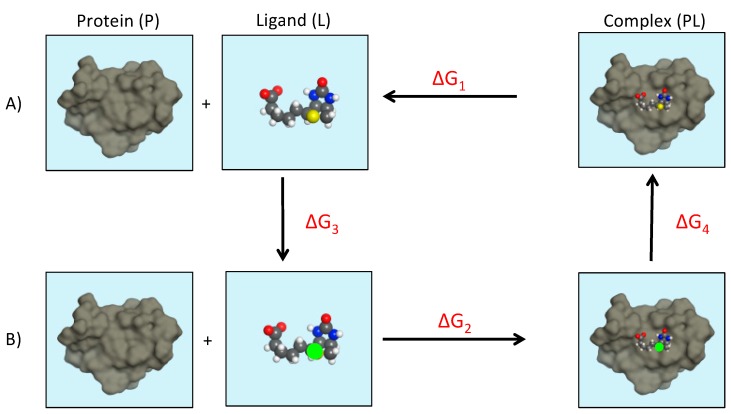
Schematic representation of a thermodynamic cycle to calculate the relative binding free energy upon a ligand modification. The binding free energy of the ligand or protein in state **A** (ΔG1) and that in state **B** (ΔG2) are the physical binding processes that need to be determined, whereas (ΔG3) and (ΔG4) (the vertical legs) indicate the unphysical transformation of ligand **A** (yellow) to ligand **B** (green). However, (ΔG3) and (ΔG4) values are more accessible to the free energy perturbation methods. According to the conservation of energy, we can derive (ΔΔG = ΔG2 − ΔG1 = ΔG4 − ΔG3).

Alchemical methods calculate the work needed to move a system from one state to another through unphysical pathways. Alchemical methods are based on a non-physical thermodynamic cycle, where the binding free energy is computed as the sum of multiple steps during which the ligand is “inserted” or “removed” from different environments, such as bound and unbound states. FEP methods can be used to investigate the effect of a change of the system (e.g., an amino acid mutation or a ligand modification) on binding free energy by applying perturbations to see if the binding affinity is improved or diminished. The relative binding free energy, it should be noted, is an indirect measure of drug potency upon the chemical change. A typical thermodynamic cycle used to calculate relative binding free energy employing FEP method is shown in Figure 9. 

FEP/MD has been successfully employed to predict the stereoselective binding of a potent modified peptide inhibitor to the HIV-1 protease [188], with the results demonstrating that the theoretical model developed can be used reliably for the prediction of relative binding affinities and should be a useful tool for the design of feasible anti-AIDS therapeutics. Interestingly, the design of HIV-1 inhibitors has emerged as an excellent testing ground for the FEP methodology in general.

The thermodynamic integration (TI) method is an alternative to the FEP method for calculating the difference in free energy between two thermodynamic states which differ from one another according to their intermolecular or intramolecular interaction potentials. In this case, the interaction potential can be expressed as a function of a coupling parameter, λ, that determines the state of the system. The free energy difference of two states is then calculated by integrating the derivative of potential energy over all coupling parameters related to a series of unphysical intermediate states [189]. 

**Figure 10:**
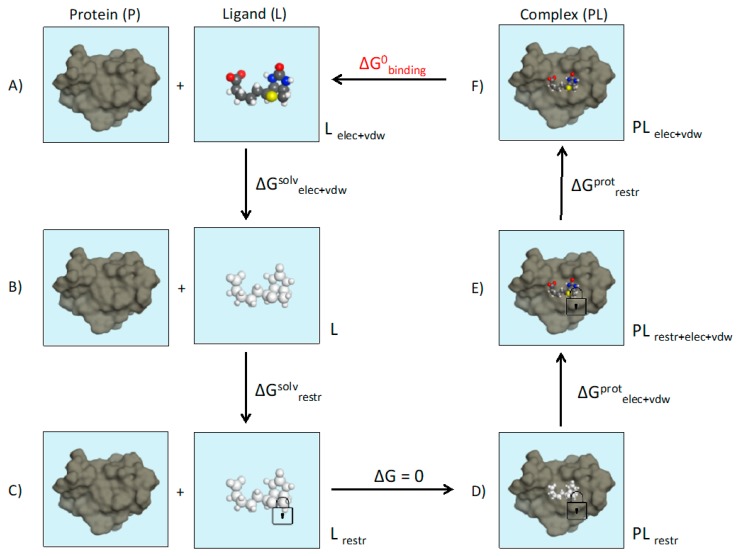
Scheme of the alchemical thermodynamic cycle used to obtain the absolute binding free energies. The restraints are represented as a lock fastening the ligand to the protein. The interacting ligand is depicted in color while the non-interacting one is transparent. The light-blue background represents the water environment. (**A**) Starting from the top-left corner, the interacting ligand (in color) is transformed to a non-interacting ligand (transparent) where its electrostatic and vdw interactions are switched off, releasing the term ΔGsolvelec+vdw. (**B**) Then a set of restraints is added to the non-interacting ligand, delivering the term, ΔGsolvrestr, which is computed analytically using the protocol proposed by Boresch [190]. (**C**) This state is equivalent to having the non-interacting ligand restrained within the protein cavity. Here the restraint is still on in order to prevent the ligand leaving the binding pocket when the interactions are scaled to zero. (**D**) To bring back the restrained non-interacting ligand in complex with protein to the interaction mode, the charges are turned back on by switching the vdW and electrostatic interactions on (ΔGprotelec+vdw). Finally, the restraints between the fully interacting ligand in complex with protein are eliminated. To calculate the absolute binding free energies (ΔGbind) we sum up the energies of a series of intermediate simulations in a closed cycle. The standard state correction (ΔG = 0) is usually added to this contribution.

Absolute binding free energies, it should be noted, have been calculated with alchemical methods for a few protein–ligand systems (see Figure 10 for details). One of the most studied macromolecular systems has been the engineered binding pocket of T4 lysozyme. Mobley et al. studied the binding of thirteen single-ring fragment-like ligands to a L99A hydrophobic T4 lysozyme cavity mutant. The computed absolute binding free energies were found to have an RMS error of 1.9 kcal/mol relative to previously determined experimental values.

In the past, applications of free energy simulations were typically limited to a very handful number of ligands that were analyzed after the experimental work. In this respect, all reports describe work that has been carried out in a retrospective manner, where ligand affinities are known and the binding conformations have usually been determined by X-ray crystallography. While retrospective studies often report good results (successful examples), the applicability of free-energy simulations to prospective work has been limited due to the lack of large-scale validation coupled with the technical challenges traditionally associated. Fortunately, in recent years more prospective free energy simulations (in some cases, combined with retrospective studies) have been published by different groups such as Janssen Pharmaceuticals [191,192,193], Jorgensen lab [179,194,195,196,197,198,199], Lovering et al. at Pfizer [200] and researches at Schrodinger and their academic collaborators [201,202] demonstrating the robustness and broad range of applicability of this approach, which can be used to drive decisions in virtual screening and lead optimization stages of drug discovery. For further reading, we refer the interested readers to other excellent recent review papers [203,204] on free energy methods. 

Although recent results from MD-based drug discovery studies are very encouraging, it ought to be borne in mind that there are still major challenges to be overcome in order to strengthen the impact of MD-based methods on drug design. Further improvement in the current FFs is required to order to achieve more accurate simulations. To date in silico drug design methods have been of vast importance in target identification and in prediction of novel drugs. In conclusion, the fusion of genomics, bioinformatics, and computational power could do wonders in improving the success of computer-aided drug design strategies, assisting in identification of new targets, determination of their structures, and the forming of a quantitative picture of the interactions between macromolecule and the ligands. Computational methods have provided a powerful toolbox for target identification, discovery, and optimization of drug candidate molecules. Information technologies coupled with statistics and chemoinformatic tools, in turn, shed light on disease mechanisms and phenotypes, revealing potential drug targets to be further validated by high throughput screening technologies. Consecutively, multiple methods allow for the prediction and characterization of binding sites through study of the dynamic nature of drug targets, identifying and optimizing new active molecular entities. Today, large databases of commercially available compounds together with ligand chemical space exploration offer drug discovery scientists an enormous volume of data with which to work. Different methods based on readily available information on the biological system under study are evolving to assist with the manipulation and processing of this data. Moreover, integration of ‘-omics’ technologies and databases may facilitate the identification of novel drug targets or the design of network-based multi-target drugs. Structure- and ligand-based methods are the most commonly used methods within the drug discovery field; however, combinatorial techniques such as proteochemometrics are gaining prominence.

In future, after the completion of human genome project, pharmacogenomics, which evaluates the effect of genes and their polymorphism on drug response will revolutionize the drug discovery and development process based on the evaluation of the different genetic markers. Furthermore, medicine will be smarter, safer, more personalized and more efficacious based on pharmacogenomics approaches [205]. Another promising example in drug discovery is that deep learning methods will become a major computer-aided drug design (CADD) approach in the near future. Although machine-learning approaches (e.g., QSAR) in modeling studies are more popular, machine intelligence approaches has been replaced with the deep learning in recent years since it can deal with complex tasks based on large, heterogeneous, and high-dimensional data sets without the need for human input [206]. Also, an emerging paradigm in modern drug discovery is of the Poly pharmacology, which is the design or use of pharmaceutical agents that act on multiple targets or disease pathways. It is generally thought that complex diseases (ie. Cancer) may require complex therapeutic approaches. Therefore, polypharmacology suggests that more effective drugs can be developed by specifically modulating multiple targets. The computational strategies plays an important role in its progress [207].

## 5. Conclusions

Large-scale MD simulation of biomolecules and biomacromolecules is an interesting and rapidly developing area that is contributing increasingly to fundamental understanding of living organisms. In the era of petascale computing today, large-scale MD simulations are having a profound impact on numerous diverse scientific endeavors, from biotechnological applications such as the fabrication of novel smart biomaterials, to DNA sequencing and the treatment of disease and development of drugs. Although several challenges lie ahead with regard to the improvement of the molecular FF and sampling of the conformational space, considering the success of these applications thus far, there is little doubt that large-scale molecular dynamics simulations will play an even more crucial and expanding role in future work in this area.

The use of computational modeling to complement experiments is helping to bridge the gap between atomic-level properties with whole-organism function, an endeavor which cannot be accomplished by either approach alone. A combination of multiple computational techniques, covering a vast range of time and size scale, is optimal for efficiently capturing information across biological scales. 

## Abbreviations

MD, Molecular dynamics; BPTI, Bovine pancreatic trypsin inhibitor; RCSB, Research Collaboratory for Structural Bioinformatics; MM, molecular mechanics; NMR, Nuclear magnetic resonance; QM, Quantum mechanics; IR, infrared spectroscopy; EVB, empirical valence bond; FF, force field; NVE, Microcanonical Ensemble; NVT, Canonical ensemble; NPT, Isotherma-isobaric ensemble; RMSD, root-mean-square deviation; CG, coarse graining, SMD, Steered molecular dynamics; US, Umbrella sampling; AFM, atomic force microscopy; WHAM, weighted histogram analysis method; PMF, potential of mean force; GPU, graphics processing units; Titania, Titanium oxide; REST, replica exchange with solution tempering; dsDNA, double-strand DNA; ssDNA, single-stranded DNA; MspA, Mycobacterium smegmatis porin A; α-HL, α-Hemolysin; CADD, Computer-aided Drug Design; LB, ligand-based; SB, structure-based; QSAR, quantitative structure–activity relationship; BOMB, biochemical and organic model builder; ADME, absorption, distribution, metabolism, and excretion/elimination; FEP, free energy perturbation; TI, thermodynamic integration.
